# Operative Dentistry

**Published:** 1875-04

**Authors:** S. J. Cobb


### ARTICLE III.

### *Operative Dentistry.*

BY S. J. COBB, D. D. S.

*Mr. President and Gentlemen of the Southern Dental Association:*

Finding my name first on the list of names composing your committee on Operative Dentistry, I feel it my duty to make some sort of reply. Not that I have anything new, or that I feel so well calculated to interest and entertain you upon this important subject, but that I wish to call attention to some extremes that a good many have reached in this department. For instance,—many of our leading dentists have abandoned the use of non-cohesive gold for filling teeth; in fact I am told that some of our colleges have become so indifferent to its use that they graduate many of their students without giving such instructions as will enable them to make a passable non-cohesive filling.

Gentlemen, when we look back over the history of dentistry and see the number of teeth that have been preserved ten, twenty, thirty and forty years, by filling them with non-cohesive gold, made into cylinders, blocks and strips, it is hard to understand how it is that so many have given up this old preparation of gold, as well as the good old ways of using it, and taking up with cohesive gold and the method of using it, which has not been in use but a few years. I hope I have not been correctly informed in regard to our colleges, for I assure you, I am slow to believe that our substantial educators are willing to give up long and well tried materials and ways of using them, until a sufficiency of time has elapsed to demonstrate clearly the superiority of new materials and methods of using them.

Cohesive gold is a very good thing in its place, but when used to the exclusion of non-cohesive it is frequently found out of its place. It is evident to me that it requires less time and skill to make a good non-cohesive filling, than it does cohesive, and as no one contends that a better filling or stopping can be made of it, in a very large majority of cases, I see no reason why any one should abandon its use. Requiring as it does the highest order of mechanism and skill to make a perfect stopping or filling with cohesive gold, and cannot be done then without true access to the parts operated upon, it is strange indeed that so many of our colleges should become so indifferent to the use of non-cohesive gold.

Requiring as stated above less time and skill to make a good filling of it than cohesive, the operation is certainly more simple and easier performed, as such should always be preferred in all plain simple operations. It seems to me that the light of our ambition should be to simplify and make plain every operation we perform on teeth, in order that all who engage in our profession should be able to do as much good as possible, but I am sorry to say that such does not seem to be the case at present. We have not only gotten a little crazy upon cohesive gold, but several other things, the mallet, rubber dam, contour fillings, machinery, and other things come in for their extreme advocacy. All of these things are good in their place, the trouble is some use them on all occasions, and condemn others that do not likewise. While the rubber dam may be indispensable in some operations, I cannot find use for it every day, neither do I find use for the mallet to drive gold into every tooth that I fill. The White and Morrison Engines are very convenient little machines to have in a dental office, but like all other good things need to be used in their place. Contour fillings well made of cohesive gold in practical cases are not only beautiful but necessary, but, I must say that practical cases are not very numerous.

I have made in the last ten years a great many contour fillings, and I am sorry to say, many of them have not proven to be quite as practical or serviceable as I would have had them. While this has been my experience, I have observed the same kind of failures made by the best of operators. Hence I have come to the conclusion that so much of this building up and restoring the crowns of teeth to their original shape and contour is not quite as practical or durable as is desirable. In my efforts to investigate these failures I find that most of them are attributable to two causes: The first is the want of a perfect adaptation of the gold to the walls of the cavities, or in other words a perfect stopping of them. The second is the want of cleanliness, resulting from the close contact of such teeth.

All teeth operated upon should be left as near self cleansing as possible, all proximal surfaces that come together after being operated upon will sooner or later decay again; as such it is important that such surfaces should be operated upon in such a way as will prevent this coming together again. I merely call attention to this extreme practice, such as, impracticable contour fillings, unnecessary use of the rubber dam, frequent heavy malleting, the use of machinery for every thing, and especially the exclusive use of cohesive gold for filling teeth, for the purpose of eliciting a little thought in this direction. A few words in regard to materials for filling teeth.

Our Dental goods manufacturers are very energetic and industrious men; they are not only providing many things for us to fill teeth with, but putting them into every conceivable shape and form that will facilitate our operations. Gold cohesive and non-cohesive is put up in various shapes and forms, so as to be suitable for all kinds of operations made by the various operators. If one should want very heavy foil he can get what is called No. 240, that is to say 240 grains in a sheet not more than 3½ inches square, then if any other should want very thin or light, he can get the same size sheet containing only 3 grains, others may get crystals or sponge gold, as well as cylinders, blocks, pellets, or corrugated foil. I have recently made some experiments with what is called Platina blocks, also foil, and I am sorry to say, my experiments were not satisfactory. I am satisfied that the Platina blocks and foil as a material for filling teeth is a failure. The blocks are intended to be cohesive but there is not enough gold on them to make them weld together perfectly. The foil worked in cylinders and strips will make a better filling than the blocks, but neither will make as good as gold. As for amalgam, there seems to be a thousand and one manufacturers, each one claiming superiority over all the rest, but with all their progression and superiority, I doubt whether we have a better amalgam to-day, than we had fifteen or twenty years ago. As for cements, there seems to be little or nothing doing in that direction; oxychloride of zinc is the best thing that I know of in the way of cement; it is a very good thing as far as it goes, but falls very far short of what we need. We want a cement of tooth color, that will stand the secretions of the mouth as well as the wear and tear from friction. I know of nothing more needed in operative dentistry at present, than a good cement, that will set quick either under or out of saliva. A cement of tooth color, impervious to the secretions oi the mouth, that will get as hard as a rock in a very short time in or out of saliva, is first what we need for filling teeth. The man that gets up such a cement, will prove to be one of the greatest benefactors that ever got up any thing to fill teeth with. When we look back over the history of cements and see how long and to what extent they have been used for other purposes, it seems strange that we have not been able to get up a better cement for filling teeth than we have. We are told that rocks or stones have been cemented together centuries ago that are as strong to day where they were cemented as elsewhere. If this is true is it not passing strange that we cannot make a cement that will stop little holes in teeth for ten or twenty years?

A few words upon the subject of exposed pulps or nerves and I will give way to others who may have something more interesting. This is an old and interesting subject, one that has been pregnant with thoughts and actions for a number of years; many ways and means have been resorted to for the purpose of protecting, and preserving this, one of the most delicate, and sensitive organs of the whole organic structure of man. In looking back over the different methods of treatment, and the different things used for covering this little sensitive organ, I find quite a number of things have been used, all resulting in failures to a very great extent.

Up to a few years ago, nothing was known better than Lead, Asbestos, Gutta-percha, Hill's Stopping, Silk, scraped Horn, or Collodion for this purpose. Some six or eight years ago Oxychloride of Zinc was brought forward, and introduced, as being superior to any and every thing used before, and for a short time swept the field as though it had been just the thing we wanted, but I am sorry to say a few years sufficed to prove that it was not all that we needed. At present we have a part composed of the Oxide of Zinc and Creasote which bids fair to supercede every thing that has been gotten up for the purpose of covering exposed pulps. This Oxide Zinc Creasote paste, as it may be called, is made by mixing Oxide of Zinc and Creasote to the consistency of very thick cream, or rather thin butter, when it should be applied to tho exposed pulps, taking care to cover the exposure well, at the same time keeping the paste away from the walls of the cavity as much as possible; as soon as this is done, fill with the Oxychloride of Zinc, as usual; after it sets, cut away as much as need be to make a metallic filling; in so doing, be careful not to cut deep enough to disturb the nerve covering; it is sometimes best to wait several days before filling with metal, but a majority of those who have been using it the longest, finish their operations the first sitting. This Oxide Zinc Creasote paste, has not been known long enough to be in general use, but a good many have been using it for two or three years with great satisfaction. As I have said before, I have no doubt but this paste will supercede every thing else that has been used for covering exposed pulps. Dr. S. J. King, of Nashville, Tenn., formerly of Petersburgh, Pa., was as far as I know the first man that ever used this paste; as such I must accord to him the credit of not only discovering this valuable remedy for the treatment of exposed pulps, but giving it to the profession.

As Dr. Arthur, our presiding officer, is present, I will call attention to his work on Prevention of Decay, as it comes under the head of Operative Dentistry, not with a spirit of criticism, for I cheerfully endorse the most of his books, but that I wish to have him explain personally his views in regard to the anticipation of decay in such cases as he would operate with an eye single to prevention.

